# Characterization of a Conjugative Hybrid Plasmid Coharboring *bla*_KPC-2_ and *bla*_IMP-4_ in a Klebsiella quasipneumoniae Clinical Isolate

**DOI:** 10.1128/spectrum.02616-22

**Published:** 2023-01-10

**Authors:** Huiyue Dong, Ziyi Liu, Zhiyao Wu, Tingting Zhang, Ziwei Xia, Yuxin Zhao, Yan Li, Jinjin Shi, Zhiqiang Wang, Ruichao Li, Shangshang Qin

**Affiliations:** a School of Pharmaceutical Sciences; Key Laboratory of Advanced Drug Preparation Technologies, Ministry of Education, Zhengzhou University, Zhengzhou, Henan, China; b Jiangsu Co-innovation Center for Prevention and Control of Important Animal Infectious Diseases and Zoonoses, College of Veterinary Medicine, Yangzhou University, Yangzhou, Jiangsu, People’s Republic of China; c Institute of Comparative Medicine, Yangzhou University, Yangzhou, Jiangsu, People’s Republic of China; Instituto de Higiene

**Keywords:** *K. quasipneumoniae*, *bla*_KPC-2_, *bla*_IMP-4_, hybrid plasmid, IncHI5 plasmid

## Abstract

Generation of hybrid MDR plasmids accelerated the evolution and transmission of resistance genes. In this study, we characterized a *bla*_KPC-2_- and *bla*_IMP-4_-coharboring conjugative hybrid plasmid constituted of an IncHI5 plasmid-like region, an IncFII(Yp)/IncFIA plasmid-like region, and a KPN1344 chromosome-like region from a clinical ST852-KL18 Klebsiella quasipneumoniae strain. The *bla*_IMP-4_ gene was captured by a novel integron In*1965*, and the *bla*_KPC-2_ gene was located on a new non-Tn*4401* group I NTE_KPC_ element. Both *bla*_KPC-2_- and *bla*_IMP-4_-containing genetic architectures were distinguished from classical structures, highlighting the constant evolution of these genetic elements.

**IMPORTANCE** The emergence of carbapenem-resistant *Enterobacterales* (CRE) that coexpress serine- and metallo-carbapenemases is a severe threat to the efficacy of ceftazidime-avibactam (CZA), which has been proven to be extremely effective against KPC-producing *Enterobacterales* strains. Our study described the cooccurrence of KPC-2, a serine β-lactamase, and IMP-4, a metallo-β-lactamase (MBL), on a conjugative hybrid plasmid from a clinical carbapenem-resistant K. quasipneumoniae strain, and it revealed an alternative route for IncHI5 plasmid to evolve by recombining with other plasmids to form a hybrid plasmid. Moreover, this hybrid plasmid can be transferred into other Klebsiella species and stably persist during passage. The propagation of two important carbapenemase genes with a new genetic background using well-evolved plasmids in the clinical setting promotes the emergence of superbugs that require careful monitoring.

## OBSERVATION

The prevalence of carbapenem-resistant *Enterobacterales* (CRE) poses a significant threat to public health. Within CRE, carbapenem-resistant Klebsiella pneumoniae (CRKP) is the predominant bacterial species (1). Besides the most commonly detected class A serine-β-lactamase KPC-2/KPC-3, metallo-β-lactamases such as NDM, VIM, and IMP were also described in CRKP conferring carbapenem resistance. Recently, the emergence of KPC-2- and NDM-1-coproducing CPKP brings challenges to the clinical treatment of CRKP; the synergistic effect of serine-β-lactamase and metallo-β-lactamase in a single isolate severely compromised the utility of ceftazidime-avibactam, meropenem-vaborbactam, and imipenem-relebactam, which are effective means to treat infections caused by KPC-producing CRKP (2). Similar to the KPC-2- and NDM-1-coproducing CPKP, current reports of *bla*_KPC_- and *bla*_IMP_-coharboring strains, including K. pneumoniae (3), Klebsiella oxytoca (4), Serratia marcescens (5), and Pseudomonas aeruginosa (6), revealed that the two different types of carbapenemase genes were carried by distinct plasmids. To date, only a nonconjugative plasmid coharboring *bla*_KPC_ and *bla*_IMP_ but without genetic feature description was identified in Raoultella ornithinolytica (7). Here, we characterized a novel conjugative hybrid plasmid named pKP18-31-IMP coharboring *bla*_KPC-2_ and *bla*_IMP-4_ in a K. quasipneumoniae strain of clinical origin.

**FIG 1:**
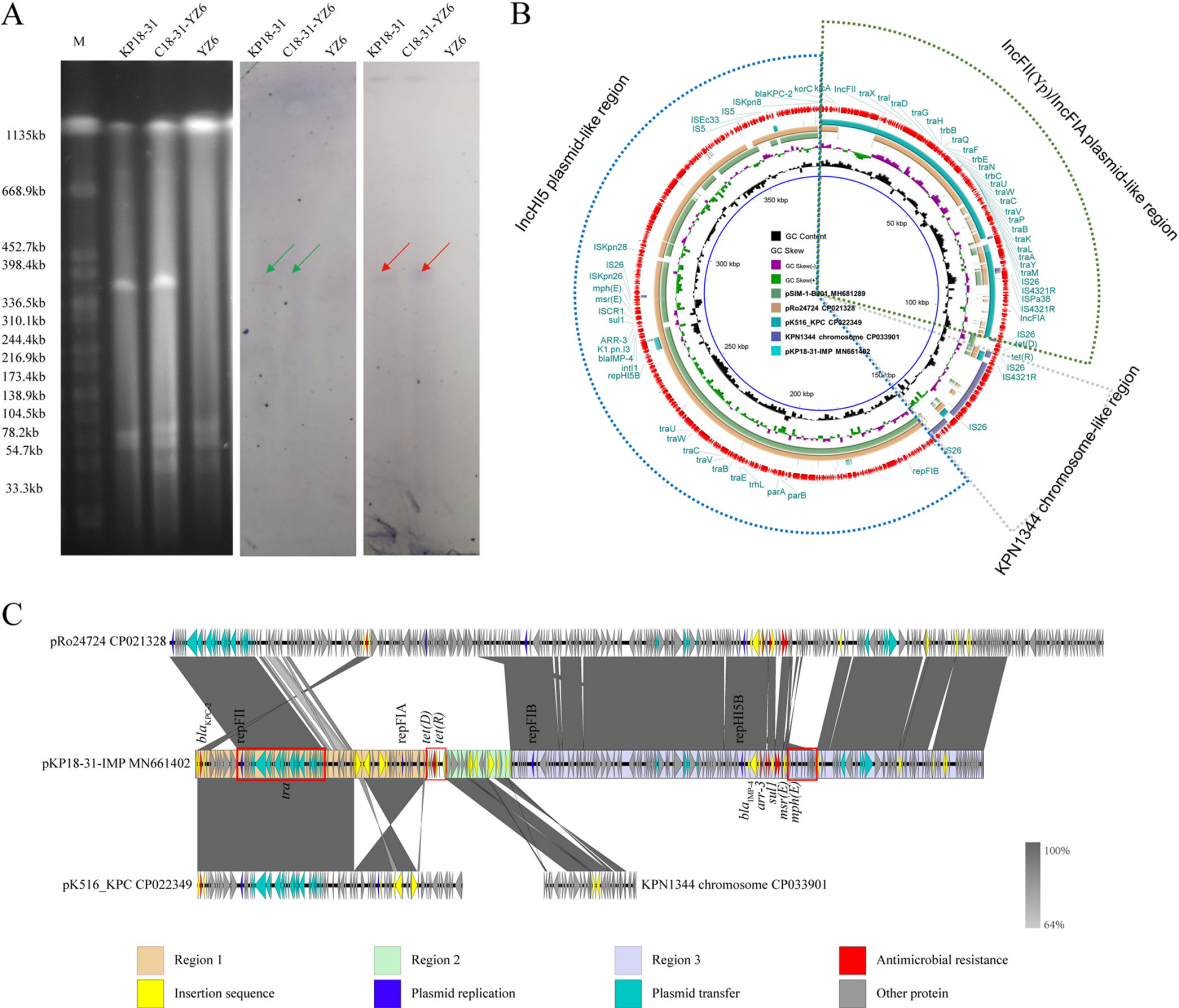
(A) PFGE of S1-digested plasmid DNA and Southern blot hybridization for *bla*_IMP-4_ (red) and *bla*_KPC-2_ (green). The black bands showed positive signals by Southern blot hybridization using the *bla*_IMP_ probe or *bla*_KPC_ probe. Lane M, Salmonella strain H9812 as a molecular marker. (B) Circular comparison between the hybrid plasmid pKP18-31-IMP and other reported similar plasmids or chromosomes. The plasmid pKP18-31-IMP at the outermost circle was used as the reference plasmid. Three circles with different sizes marked in blue, green, and gray dash lines represent the different genetic modules of pKP18-31-IMP. The circular map was generated using the BLAST Ring Image Generator (BRIG). (C) Linear alignment of plasmid pKP18-31-IMP with plasmid pRo24724 and plasmid pK516_KPC. The linear map was generated using EasyFig.

The clinical strain KP18-31 was isolated from the blood culture of a 12-year-old female patient with severe aplastic anemia in 2018 (Table 1). By using BIGSdb (https://pubmlst.org/) and Kleborate software (8, 9), the *in silico* species identification results showed that KP18-31 belonged to K. quasipneumoniae, which has been reported as an emerging pathogen in 2004 that can cause bloodstream infections in healthy individuals (10). For measuring the antimicrobial susceptibility profiles, agar dilution (for fosfomycin) and broth microdilution methods according to the Clinical and Laboratory Standards Institute (CLSI) guidelines were recruited (11). KP18-31 was resistant to a variety of antibiotics, including meropenem and aztreonam (Table 1). Whole-genome sequencing (WGS) results indicated that KP18-31 carried both *bla*_KPC-2_ and *bla*_IMP-4_. In order to investigate the transferability of *bla*_KPC-2_ and *bla*_IMP-4_, KP18-31 was used as the donor strain, and ST11 K. pneumoniae HS11286YZ6 was used as the recipient strain for conjugation assay (12). According to conjugation assay, both *bla*_KPC-2_ and *bla*_IMP-4_ were successfully transferred into the recipient strain HS11286YZ6. S1 pulsed-field gel electrophoresis (PFGE) and Southern hybridization revealed that the *bla*_KPC-2_ and *bla*_IMP-4_ genes were colocated on an ~370-kb plasmid (Fig. 1A) in KP18-31 and the corresponding transconjugant, suggesting that the plasmid simultaneously encoding two important carbapenemases was able to spread horizontally. Furthermore, the conjugation frequency (the number of transconjugants/total number of recipients) of this plasmid was 2.0 × 10^−5^, and stability tests demonstrated that it could be stably maintained in the transconjugant at 150th generations (66.7%) without antibiotic selection, indicating it was well adapted to the host (13).

**TABLE 1:** Antimicrobial susceptibility profiles of K. quasipneumoniae KP18-31

Antimicrobial category	Antimicrobial agent	MIC (μg/mL) of:^*a*^		
		KP18-31^*b*^	C18-31-YZ6^*c*^	YZ6^*d*^
Penicillins	Ampicillin	>256	>256	>256
Antipseudomonal penicillins with β-lactamase inhibitors	Piperacillin-tazobactam	128	>512	512
Nonextended spectrum cephalosporins	Cefazolin	>256	>256	>256
Extended-spectrum cephalosporins	Ceftazidime	>64	>64	8
	Cefepime	>256	>256	128
Carbapenems	Imipenem	32	32	0.5
	Meropenem	32	16	0.06
Monobactams	Aztreonam	64	>64	64
Fluoroquinolones	Ciprofloxacin	<0.015	4	8
Aminoglycosides	Gentamicin	<0.25	1	0.5
	Amikacin	0.5	1	1
Phenicols	Chloramphenicol	16	4	4
Tetracyclines	Doxycycline	>128	64	4
Phosphonic acid	Fosfomycin	<1	256	256
Glycylcyclines	Tigecycline	0.25	0.25	1
Polymyxin C	Colistin	<0.125	<0.125	1
Ceftazidime-avibactam	Ceftazidime-avibactam	128/4	0.5/4	0.5/4
Aztreonam-avibactam	Aztreonam-avibactam	0.5/4	0.5/4	0.5/4

a MICs were determined by broth or agar dilution as proposed by CLSI and interpreted using its guidelines.

b Donor strain.

c Corresponding transconjugant.

d Recipient strain.

To resolve the complete plasmid sequence of KP18-31, the whole-genome DNA of strain KP18-31 was extracted (Wizard genomic DNA purification kit) and used for WGS by Illumina HiSeq2500 platform (San Diego, CA, United States) and PacBio RS platform (Pacific Biosciences, Menlo Park, CA, USA). Multilocus sequence typing (MLST), the distribution of plasmid replicons, antimicrobial resistance (AMR) genes, and insertion sequences were confirmed by online tools (https://cge.food.dtu.dk/services/) and Kleborate software (8). Strain KP18-31, which was assigned to ST852 and KL18 based on Kleborate analysis, was found to harbor one chromosome of 5,157,396 bp (GenBank accession no. CP045641) and three plasmids named pKP18-31-IMP (GenBank accession no. MN661402), pKP18-31-2 (GenBank accession no. MN661403), and pKP18-31-3 (GenBank accession no. MN661404) of 377,346 bp, 119,801 bp, and 107,000 bp, respectively. Resistome analysis revealed four AMR genes, including *oqxA*, *oqxB*, *bla*_OKP-B-2_, and *fosA*, were detected on the chromosome, while the remaining eight AMR genes, including *bla*_KPC-2_, *bla*_IMP-4_, *msr*(E), *mph*(E), *arr-3*, *sul1*, *tet*(D), and *tet*(R) were identified on plasmid pKP18-31-IMP, and no AMR gene was carried by the other two smaller plasmids, pKP-18-31-2 and pKP-31-3, which belonged to IncFII(K) and untypeable plasmids, respectively.

Plasmid pKP18-31-IMP in this study simultaneously possessed four plasmid replicons, including IncFII(Yp), IncFIA, IncFIB-like, and IncHI5B. Previously, *bla*_IMP_-positive plasmids carrying multiple plasmid replicons have also been identified in species other than K. quasipneumoniae, such as an IncFII/IncU/IncFIB/IncHI5B-type plasmid pRo24724 (GenBank accession no. CP021328) found in Raoultella ornithinolytica and an IncHIB/IncFIA/IncR-type plasmid pKOX75251 (GenBank accession no. CP065475) found in Klebsiella michiganensis (7, 14). Given that IncFIB-like and IncHI5B replication genes are used to distinguish IncHI5 plasmids (15), the presence of two extra replication genes suggested that the formation of plasmid pKP18-31-IMP might have been driven by the recombination of an IncHI5 plasmid with another plasmid. A BLASTN search against the NCBI nucleotide database revealed that pKP18-31-IMP displayed 80% query coverage and 99.97% nucleotide identity to plasmid pRo24724 (GenBank accession no. CP021328) from the R. ornithinolytica strain, 59% query coverage and 99.95% nucleotide identity to IncHI5 plasmid pSIM-1-BJ01 (GenBank accession no. MH681289) from the K. pneumoniae strain, and 31% query coverage and 99.81% nucleotide identity to IncFII(Yp)/IncFIA plasmid pK516_KPC (GenBank accession no. CP022349) from the K. michiganensis strain. Detailed sequence analysis of pKP18-31-IMP showed it was a cointegrate plasmid consisting of an ~226-kb pRo24724-derived module (IncHI5 plasmid-like region, region 3), an ~110-kb pK516_KPC-derived module (IncFII(Yp)/IncFIA plasmid-like region, region 1), and an ~28-kb segment contained open reading frames (ORFs) coding for products related to a bifunctional protein (zinc-containing alcohol dehydrogenase and quinone oxidoreductase), beta-galactosidase LacZ, lactose permease LacY, and maltoporin; it shares high identity (88% query coverage and 100% nucleotide identity) with the corresponding fragment in the chromosome of K. pneumoniae strain KPN1344 (GenBank accession no. CP033901) (KPN1344 chromosome-like region, region 2) (Fig. 1B and C). Notably, linear comparison revealed that a 34,216-bp region carrying genes related to plasmid replication and transfer (within the red box) organized as *repFII-finO-traXIDGH-trbB-traQF-trbE-traNCUW-trbI-traCVPAKELAYW* in pKP18-31-IMP was shared by pRo24724 and pK516_KPC, suggesting that homologous recombination within this region might have led to the formation of a pRo24724:pK516_KPC hybrid (Fig. 1C). Moreover, inversion of a 30,235-bp region in pK516_KPC-derived region 1, integration of an ~28-kb segment highly homologous to the KPN1344 chromosome (region 2), and an ~5-kb IS*26*-flanked segment carrying *tet*(*R*) and *tet*(*D*) genes between regions 1 and 2, and rearrangement of a 12,539-bp (within the red box) segment in pRo24724-derived region 3, further suggested that the formation of pKP18-31-IMP has undergone complex and multiple recombination processes based on pRo24724-like and pK516_KPC-like structures. Recently, we have identified another IncHI5-like plasmid, pC39-334kb, and inferred that the plasmid was evolved from the pNDM-1-EC12-like IncHI5 plasmids (16). These findings jointly highlight the promising evolutionary potential of IncHI5 plasmids, as they may lead to the formation of novel multidrug resistance plasmids and further expand their host range (17).

**FIG 2:**
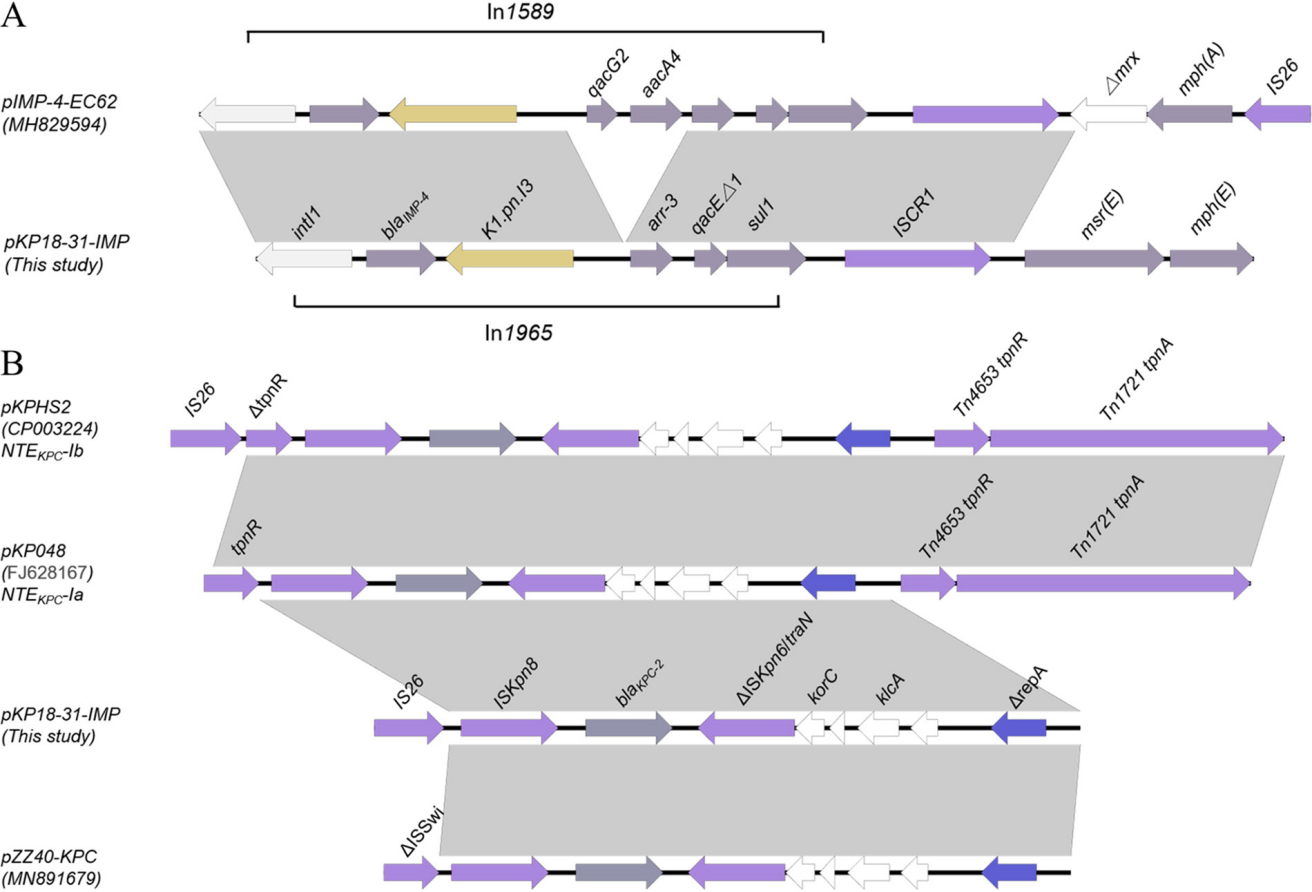
(A) Linear comparison of genetic environment of *bla*_IMP-4_ between pKP18-31-IMP and pIMP-4-EC62. (B) Linear comparison of the genetic environment of *bla*_KPC-2_ among pKPHS2, pKP048, pKP18-31-IMP, and pZZ40-KPC. The plasmid comparison figure was generated by EasyFig.

The *bla*_IMP-4_ gene was identified within class 1 integron with the *intI1*-*bla*_IMP-4_*-K1.pn.I3-arr-3-qacE*Δ1*-sul1* genetic array, which was given a new integron number, In*1965*, by the INTEGRALL database. The variable region of In*1965* was interrupted by the insertion of group II intron *K1.pn.I3*, downstream of the *attC* site (also named the 59-base element) of the *bla*_IMP-4_ gene cassette. Compared to the In*1589* reported previously (18), the two integrons were identical except for the loss of two genes, *qacG2* and *aacA4* (Fig. 2A). The *bla*_IMP-4_ gene was usually captured by a class 1 integron, including In*1589*, In*823*, In*1310*, In*809*, etc. (19). In this study, we described a novel *bla*_IMP-4_-*K1.pn.I3*-harboring class 1 integron. As previously reported, *bla*_IMP-4_-*K1.pn.I3* has been regarded as the most common structure in IMP-4-producing isolates in China (18). Previous studies showed that transposons such as Tn*6017* and Tn*1696* are important vectors mediating the spread of *bla*_IMP_ in *Enterobacterales*. Whether transposons can promote the spread of the *bla*_IMP-4_-harboring integron-carrying In*1965* requires further investigation.

The *bla*_KPC-2_ gene was located on a non-Tn*4401* element with the structure of IS*26*-IS*Kpn8*-*bla*_KPC-2_-ΔIS*Kpn6*/*traN*-*korC*-*klcA*-Δ*repA*. Compared to the classical NTEKPC-Ib structure in pKPHS2 (GenBank accession no. CP003224) (20), *ΔtnpR* upstream of *bla*_KPC-2_ and Tn*4653 tnpR* and Tn*1721 tnpR* downstream of *bla*_KPC-2_ were absent in the *bla*_KPC-2_ genetic context of pKP18-31-IMP. Alternatively, this genetic array also shared high homology to the NTEKPC-Ia structure in pKP048 (GenBank accession no. FJ628167) (Fig. 2B). In addition, we found that the genetic environment of *bla*_KPC-2_ in plasmid pZZ40-KPC (GenBank accession no. MN891679) had 91% query coverage compared with that in plasmid pKP18-31-IMP. However, the main difference between them is the upstream region of *bla*_KPC-2_ with different genes (IS*26* and ΔIS*Swi*) (Fig. 2B). These findings provided substantial evidence that the *bla*_KPC-2_ gene was located on a new non-Tn*4401* element in this study. Since the different genetic organizations containing *bla*_KPC-2_ were detected from the traditional Tn*4401* in 2009 (21), various mutations around *bla*_KPC_ were reported (22). This novel structure could be assigned to the group I NTE element because no insertion sequence was observed upstream of *bla*_KPC-2_ (20). However, unlike group I NTE elements that usually have a *tnpR* gene upstream of IS*Kpn8*, the *tnpR* gene is missing in the new element, suggesting that the formation of the structure may undergo various recombination events.

In conclusion, we characterized a *bla*_IMP-4_- and *bla*_KPC-2_-coharboring conjugative hybrid plasmid pKP18-31-IMP from K. quasipneumoniae. Three regions of an IncHI5 plasmid-like region, an IncFII(Yp)/IncFIA plasmid-like region, and a KPN1344 chromosome-like region construct of the basic structure of the plasmid highlight the strong evolutionary ability of IncHI5 plasmids. Both *bla*_IMP-4_ and *bla*_KPC-2_ were surrounded by novel genetic contexts, which differ to some extent from that reported previously. The spread of two critical AMR genes with the novel genetic contexts using the hybrid plasmid as the vehicle in clinical settings will facilitate the emergence of superbugs, which requires stringent surveillance.

### Data availability.

The complete sequences of KP18-31 were submitted to the NCBI GenBank database with the following accession numbers: chromosome, CP045641; pKP18-31-IMP, MN661402; pKP18-31-2, MN661403; and pKP18-31-3, MN661404.

## References

[1] Lai YC, Lu MC, Hsueh PR. 2019. Hypervirulence and carbapenem resistance: two distinct evolutionary directions that led high-risk *Klebsiella pneumoniae* clones to epidemic success. Expert Rev Mol Diagn 19:825–837. doi:10.1080/14737159.2019.1649145. 31343934

[2] Gao H, Liu YD, Wang RB, Wang Q, Jin LY, Wang H. 2020. The transferability and evolution of NDM-1 and KPC-2 co-producing *Klebsiella pneumoniae* from clinical settings. Ebiomedicine 51:102599. doi:10.1016/j.ebiom.2019.102599. 31911273 PMC6948161

[3] Wang Y, Cao W, Zhu X, Chen Z, Li L, Zhang B, Wang B, Tian L, Wang F, Liu C, Sun Z. 2012. Characterization of a novel *Klebsiella pneumoniae* sequence type 476 carrying both *bla*_KPC-2_ and *bla*_IMP-4_. Eur J Clin Microbiol Infect Dis 31:1867–1872. doi:10.1007/s10096-011-1512-7. 22271301

[4] Wang J, Yuan M, Chen H, Chen X, Jia Y, Zhu X, Bai L, Bai X, Fanning S, Lu J, Li J. 2017. First report of *Klebsiella oxytoca* strain simultaneously producing NDM-1, IMP-4, and KPC-2 carbapenemases. Antimicrob Agents Chemother 61:e00877-17. doi:10.1128/AAC.00877-17. 28674063 PMC5571352

[5] Silva KE, Cayo R, Carvalhaes CG, Patussi Correia Sacchi F, Rodrigues-Costa F, Ramos da Silva AC, Croda J, Gales AC, Simionatto S. 2015. Coproduction of KPC-2 and IMP-10 in carbapenem-resistant *Serratia marcescens* isolates from an outbreak in a Brazilian teaching hospital. J Clin Microbiol 53:2324–2328. doi:10.1128/JCM.00727-15. 25878341 PMC4473237

[6] Martinez T, Vazquez GJ, Aquino EE, Ramirez-Ronda R, Robledo IE. 2012. First report of a *Pseudomonas aeruginosa* clinical isolate co-harbouring KPC-2 and IMP-18 carbapenemases. Int J Antimicrob Agents 39:542–543. doi:10.1016/j.ijantimicag.2012.02.009. 22521765

[7] Zheng B, Zhang J, Ji J, Fang Y, Shen P, Ying C, Lv J, Xiao Y, Li L. 2015. Emergence of *Raoultella ornithinolytica* coproducing IMP-4 and KPC-2 carbapenemases in China. Antimicrob Agents Chemother 59:7086–7089. doi:10.1128/AAC.01363-15. 26282422 PMC4604418

[8] Wick RR, Heinz E, Holt KE, Wyres KL. 2018. Kaptive Web: user-friendly capsule and lipopolysaccharide serotype prediction for *Klebsiella* genomes. J Clin Microbiol 56:e00197-18. doi:10.1128/JCM.00197-18. 29618504 PMC5971559

[9] Jolley KA, Bray JE, Maiden MCJ. 2018. Open-access bacterial population genomics: BIGSdb software, the PubMLST.org website and their applications. Wellcome Open Res 3:124. doi:10.12688/wellcomeopenres.14826.1. 30345391 PMC6192448

[10] Imai K, Ishibashi N, Kodana M, Tarumoto N, Sakai J, Kawamura T, Takeuchi S, Taji Y, Ebihara Y, Ikebuchi K, Murakami T, Maeda T, Mitsutake K, Maesaki S. 2019. Clinical characteristics in blood stream infections caused by *Klebsiella pneumoniae*, *Klebsiella variicola*, and *Klebsiella quasipneumoniae*: a comparative study, Japan, 2014–2017. BMC Infect Dis 19:946. doi:10.1186/s12879-019-4498-x. 31703559 PMC6842162

[11] Clinical and Laboratory Standards Institute. 2018. Performance standards for antimicrobial susceptibility testing: twenty-fourth informational supplement. CLSI M100-S28. Clinical and Laboratory Standards Institute, Wayne, PA.

[12] Chen R, Liu Z, Xu P, Qi X, Qin S, Wang Z, Li R. 2021. Deciphering the epidemiological characteristics and molecular features of *bla*_KPC-2_- or *bla*_NDM-1_-positive *Klebsiella pneumoniae* isolates in a newly established hospital. Front Microbiol 12:741093. doi:10.3389/fmicb.2021.741093. 34858362 PMC8631570

[13] Liu Z, Wang Z, Lu X, Peng K, Chen S, He S, Li R. 2021. Structural diversity, fitness cost, and stability of a Bla(NDM-1)-bearing cointegrate plasmid in *Klebsiella pneumoniae* and *Escherichia coli*. Microorganisms 9:2435. doi:10.3390/microorganisms9122435. 34946035 PMC8708245

[14] Li X, He J, Jiang Y, Peng M, Yu Y, Fu Y. 2021. Genetic characterization and passage instability of a hybrid plasmid co-harboring *bla*_IMP-4_ and *bla*_NDM-1_ reveal the contribution of insertion sequences during plasmid formation and evolution. Microbiol Spectr 9:e0157721. doi:10.1128/Spectrum.01577-21. 34908434 PMC8672901

[15] Liang Q, Yin Z, Zhao Y, Liang L, Feng J, Zhan Z, Wang H, Song Y, Tong Y, Wu W, Chen W, Wang J, Jiang L, Zhou D. 2017. Sequencing and comparative genomics analysis of the IncHI2 plasmids pT5282-mphA and p112298-catA and the IncHI5 plasmid pYNKP001-dfrA. Int J Antimicrob Agents 49:709–718. doi:10.1016/j.ijantimicag.2017.01.021. 28390961

[16] Liu Z, Chen R, Xu P, Wang Z, Li R. 2021. Characterization of a *bla*_NDM-1_-bearing IncHI5-like plasmid from *Klebsiella pneumoniae* of infant origin. Front Cell Infect Microbiol 11:738053. doi:10.3389/fcimb.2021.738053. 34660344 PMC8517479

[17] Xie MM, Li RC, Liu ZH, Chan EWC, Chen S. 2018. Recombination of plasmids in a carbapenem-resistant NDM-5-producing clinical *Escherichia coli* isolate. J Antimicrob Chemother 73:1230–1234. doi:10.1093/jac/dkx540. 29373691

[18] Zhu Y, Zhang W, Schwarz S, Wang C, Liu W, Chen F, Luan T, Liu S. 2019. Characterization of a *bla*_IMP-4_-carrying plasmid from *Enterobacter cloacae* of swine origin. J Antimicrob Chemother 74:1799–1806. doi:10.1093/jac/dkz107. 30879063

[19] Liu W, Dong H, Yan T, Liu X, Cheng J, Liu C, Zhang S, Feng X, Liu L, Wang Z, Qin S. 2021. Molecular characterization of *bla*_IMP-4_-carrying *Enterobacterales* in Henan Province of China. Front Microbiol 12:626160. doi:10.3389/fmicb.2021.626160. 33679645 PMC7925629

[20] Chen L, Mathema B, Chavda KD, DeLeo FR, Bonomo RA, Kreiswirth BN. 2014. Carbapenemase-producing *Klebsiella pneumoniae*: molecular and genetic decoding. Trends Microbiol 22:686–696. doi:10.1016/j.tim.2014.09.003. 25304194 PMC4365952

[21] Shen P, Wei Z, Jiang Y, Du X, Ji S, Yu Y, Li L. 2009. Novel genetic environment of the carbapenem-hydrolyzing beta-lactamase KPC-2 among *Enterobacteriaceae* in China. Antimicrob Agents Chemother 53:4333–4338. doi:10.1128/AAC.00260-09. 19620332 PMC2764158

[22] Huang QS, Liao W, Xiong Z, Li D, Du FL, Xiang TX, Wei D, Wan LG, Liu Y, Zhang W. 2021. Prevalence of the NTE(KPC)-I on IncF plasmids among hypervirulent *Klebsiella* pneumoniae isolates in Jiangxi Province, South China. Front Microbiol 12:622280. doi:10.3389/fmicb.2021.622280. 34234750 PMC8256152

